# Furuncular myiasis by *Cordylobia anthropophaga* in three Italian children: First case series and implications for non-endemic regions

**DOI:** 10.1371/journal.pntd.0013535

**Published:** 2026-05-11

**Authors:** Livia Mancinelli, Marika Guercio, Gianluca Vrenna, Manuela Onori, Claudio De Liberato, Adele Magliano, Andrea Diociaiuti, Massimiliano Raponi, Carlo Federico Perno, May El Hachem, Paola Bernaschi

**Affiliations:** 1 Unit of Microbiology and Diagnostic Immunology, Bambino Gesù Children’s Hospital, IRCCS, Rome, Italy; 2 Department of Pediatric Hematology and Oncology and of Cell and Gene Therapy, Bambino Gesù Children’s Hospital, IRCCS, Rome, Italy; 3 Istituto Zooprofilattico Sperimentale del Lazio e della Toscana “M. Aleandri”, Rome, Italy; 4 Dermatology Unit, Genodermatosis Research Unit, Translational Paediatrics and Clinical Genetics Research Area, Bambino Gesù Children’s Hospital, IRCCS, Rome, Italy; 5 Medical Direction, Bambino Gesù Children’s Hospital, IRCCS, Rome, Italy‌‌; Cyprus International University: Uluslararasi Kibris Universitesi, CYPRUS

## Abstract

*Cordylobia anthropophaga,* commonly known as the *tumbu* fly, is a leading cause of cutaneous myiasis in sub-Saharan Africa. This condition is characterized by a papulopustular lesion that evolves in a painful boil-like nodule with central ulceration. Human infestations typically occur on covered body parts, due to the fly’s habit of laying eggs on damp clothing. This report describes the first cluster of three pediatric cases of furuncular myiasis caused by *C. anthropophaga*, diagnosed at the Bambino Gesù Children’s Hospital (Rome, Italy) between April 2024 and April 2025. All patients had recent travel history to endemic African regions and presented with cutaneous nodules, some accompanied by systemic signs such as fever or regional lymphadenopathy. Diagnosis was confirmed through clinical evaluation and morphological identification of the extracted larvae. Treatment consisted of occlusive therapy to facilitate larval expulsion or surgical extraction of the maggots and systemic antibiotics. One patient also exhibited a family cluster, with larval extraction from a parent. Laboratory results were largely unremarkable, consistent with localized infection. These cases highlight the growing relevance of imported myiasis in non-endemic countries like Italy, due to increased international travel and potential climate-driven changes in vector distribution. Enhanced awareness among travelers and healthcare providers, together with proactive public health measures, is crucial. By documenting these cases, we hope to contribute to the understanding of emerging parasitic diseases in non-endemic regions and reinforce the need for vigilance in this time of global environmental changes.

## Author summary

Furuncular myiasis is a parasitic skin infestation caused by fly larvae that penetrate the skin and develop within the dermis, producing painful boil-like nodules with a central opening. *Cordylobia anthropophaga*, also known as the tumbu fly, is a common cause of furuncular myiasis in sub-Saharan Africa, but cases are increasingly reported in non-endemic regions due to international travel and changing environmental conditions.

We describe the first published Italian pediatric case series of furuncular myiasis caused by *C. anthropophaga*, diagnosed in three children between April 2024 and April 2025. All patients had a recent history of travel to endemic areas and presented with inflammatory nodules on the back, initially suspected to be bacterial infections or insect bites. Diagnosis was confirmed by dermoscopy and parasitological identification of the larvae.

Our report highlights the importance of considering imported myiasis in children returning from endemic regions with persistent nodular skin lesions, even in high-income, non-endemic countries. Increased awareness among pediatricians, dermatologists, infectious disease specialists, and primary care physicians can prevent misdiagnosis, unnecessary antibiotic use, and delayed treatment. From a One Health perspective, surveillance, traveler education, and attention to the potential impact of climate and environmental changes are essential to limit the spread of this parasitic disease beyond its traditional geographic range.

## Introduction

*Cordylobia anthropophaga* (Blanchard, 1893; Diptera: Calliphoridae), also named as the *tumbu* fly, is one of the most frequent causes of cutaneous myiasis in sub-Saharan Africa [1]. The infestation typically presents as a papulopustular lesion with a central opening, which evolves over several days into a painful inflammatory nodule [2].

The biology of this fly is well described in the literature. Female flies live for about two weeks and lay their eggs in sand, soil contaminated with urine or faeces, and damp clothes that are being dried outdoors and not ironed.

At ambient temperature, the larvae usually hatch on the third day and remain under the surface of the sand, sensitive to heat and vibration, waiting for a host. Larvae are able to penetrate undamaged skin, and penetration is barely noticed. During the first two days, a small papule develops at the affected skin site, accompanied by mild itching or tingling. By day three, the second larval stage appears and the papule has increased in size, with surrounding erythema and persistent irritation over several days. The larvae are usually discovered at the end of the first week of illness, when the early third stage has been reached, and the pain suddenly returns with great severity. At this stage, the larva feeds actively on serous fluids and tissue cells, using its mouth hooks and cytolytic salivary secretions. This induces a lytic inflammatory reaction with hardened, darkened, and pressure-sensitive surrounding tissue. The initially watery exudate becomes serous and may contain blood or larval excrement [3]. Local lymphadenopathy is common [4], and systemic symptoms such as fever and general malaise may occur [5].

**Fig 1:**
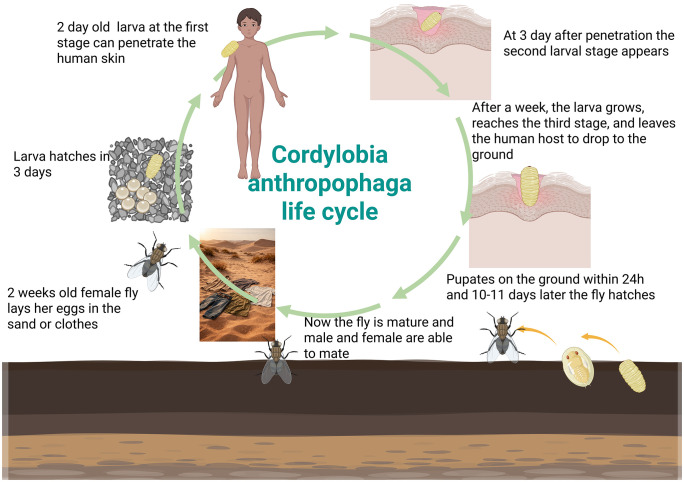
Timeline of *Cordylobia anthropophaga* life cycle. Created in BioRender. Vrenna, G. (2026) https://BioRender.com/u29b796. Publication under an open-access license (CC BY 4.0) is permitted under the BioRender Academic Publication License.

Human infestations predominantly affect covered areas of the body, as eggs are commonly laid on clothing left to dry outdoors, allowing larvae to penetrate directly into the underlying skin once the garments are worn. After completing development, the larva exits the lesion, falls to the ground, and pupates within 24 hours. The adult fly emerges after 10–11 days (Fig 1).

*Tumbu* fly myiasis is common in many sub-Saharan African countries [1–5], but cases are increasingly reported in non-endemic areas due to international travel, particularly among returning travelers. Although most European cases are imported, rare autochthonous infections have been described [6]. Children appear especially vulnerable, probably because of closer contact with contaminated soil or clothing [5,7]. In non-endemic countries, limited awareness leads to frequent misdiagnosis as bacterial abscesses or furunculosis, with delayed diagnosis and inappropriate treatment [8,9]. Familiarity with the clinical and dermoscopic features of cutaneous myiasis is therefore essential, especially in patients with a relevant travel history [2,4,10].

## Methods

### Study design and patients

This study is a retrospective descriptive case series of pediatric patients diagnosed with furuncular myiasis caused by *C. anthropophaga* in Italy between April 2024 and April 2025. Three children were referred to our institution after recent travel to sub-Saharan Africa and were evaluated for cutaneous lesions suspicious for parasitic infestation. Demographic data, travel history, clinical presentation, diagnostic procedures, management, and outcomes were collected from medical records.

### Clinical and laboratory assessment

All patients underwent a complete clinical and dermatological examination. Lesions were evaluated for number, location, size, presence of a central punctum, and associated symptoms such as pain, pruritus, or discharge. When clinically indicated, laboratory investigations were performed to exclude alternative diagnoses or associated infections, including complete blood count and inflammatory markers. In patients presenting with systemic symptoms, additional investigations were carried out according to clinical judgment.

### Parasitological identification and management

Larvae were extracted using standard non-invasive or minimally invasive techniques. When needed to facilitate larval emergence, an occlusive agent such as petroleum jelly was applied. Alternatively, larvae were carefully removed with fine forceps under aseptic conditions, avoiding excessive pressure or large incisions to minimize the risk of rupture and tissue damage. Extracted specimens were examined both macroscopically and microscopically, and identification was based on morphological features, including body spines and posterior spiracles, consistent with *C. anthropophaga* larvae. Following removal, lesions were cleaned and monitored until complete resolution.

### Ethics statement

The study is based on retrospective analysis of anonymized clinical data. Written informed consent for the use of clinical information and publication of images was obtained from the parents or legal guardians of all patients. According to OPBG institutional policy, formal ethics committee approval was not required for retrospective descriptive case series using fully anonymized data.

## Results

### Case series

Three pediatric patients were diagnosed with furuncular myiasis caused by *C. anthropophaga*. All children had recently returned to Italy after travel to sub-Saharan Africa. None had a previous history of immunodeficiency or chronic disease. All patients presented with painful, erythematous, boil-like cutaneous nodules, each characterized by a central opening. In all cases, lesions were located on the back. The number of lesions varied among patients, with each lesion corresponding to a single larva.

**Patient 1.** A two-year-old male, living in the Dakar area of Senegal, returned to Italy with furuncular lesions on his back. The lesions were progressive, accompanied by redness and swelling in absence of history of fever or vomiting. When the child was admitted to the Emergency Department of the Bambino Gesù Children’s Hospital in April 2024, his mother reported that the lesions had appeared five days earlier, when he was still in Africa, and had been treated at home with topical petroleum jelly. She also observed the discharge of a fly maggot from one of the lesions. The child usually plays in the garden and at the beach. They have a cat at home. No other family members have reported similar lesions.

**Fig 2:**
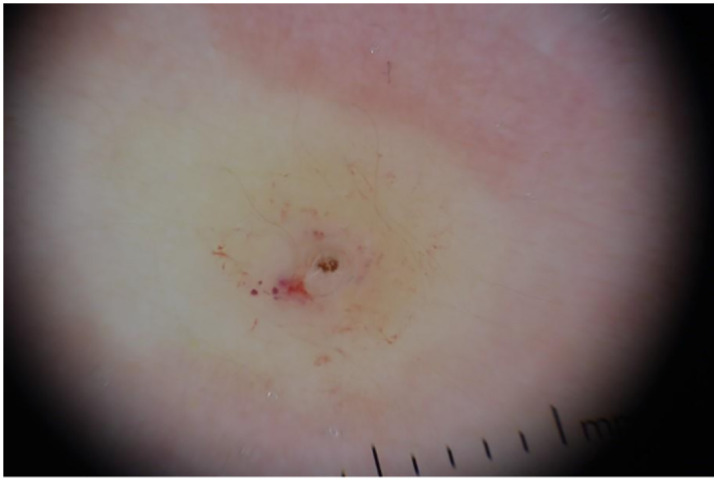
The dermatoscopic observation of one of the lesions showed a yellowish color, black dots, vascular pattern and the bird’s feet sign corresponding to the breathing spiracles of the larva (polarized dermoscopy, 10X magnification). Clinical image used with written permission of the copyright holder(s) for publication under the Creative Commons Attribution 4.0 (CC BY 4.0) license. Written informed consent for publication of clinical images was obtained from the patient’s parents/legal guardians.

The patient presented two swelling nodules on the back. Each one had a 2 cm diameter with ill-demarcated edges and a central ulceration. There was little tenderness and scanty serous exudates. Dermoscopy showed a yellowish color, black dots and the “bird’s feet sign”, a specific dermoscopic feature of this disease (Fig 2).

On physical examination, the child presented good overall condition: perfused, nourished, and well hydrated. No regional or systemic lymphadenopathy was observed, the child presented normal breathing sounds and regular heart rate and rhythm. The abdomen was soft and non-tender with normal bowel sounds. Blood tests showed only a mild increase in eosinophil percentage (WBC 9,950/µl, Neutrophils 3,670/µl (36.9%), Lymphocytes 4,210/µl (42.3%), Eosinophils 840/µl (8.4%), Hb 12.6 g/dl) and normal inflammatory markers (CRP 0.08 mg/dL, LDH 322 U/L). Clinical diagnosis of furuncular myiasis was given.

On the same day, surgical removal of the larva in deep sedation was performed due to patient age. The skin was incised, and a live and mobile maggot, approximately 10 mm long and 4 mm wide, was successfully extracted. The larva was preserved in a solution of 10% KOH and 90% ethanol sequentially and sent to the parasitology department.

After surgery, systemic antibiotic treatment (amoxicillin-clavulanic acid 50 mg per kg b.i.d.) was administered and the child was discharged with an indication to continue oral antimicrobial therapy for one week.

**Patient 2.** A seven-year-old girl living in Anzio, Italy, presented to the Emergency Department of the Bambino Gesù Children’s Hospital in February 2025 with a furuncular lesion on her back and another on the scalp, accompanied by periorbital swelling. Her mother reported no history of fever or systemic symptoms. The child had recently returned from a vacation in Kenya, one week before, during which no malaria prophylaxis was undertaken. She had a medical history of bronchial asthma.

On examination, the patient was in good general condition: alert, well hydrated, and without fever. A furuncular lesion with crust on top was observed in the interscapular region, which the father had attempted to squeeze prior the evaluation to drain pus. A second similar lesion was noted on the right parietal scalp region. There was mild edema of the nasal bridge and upper eyelids, as well as bilateral latero-cervical and inguinal lymphadenopathy. Cardiopulmonary and abdominal examinations were unremarkable.

Laboratory tests revealed a normal complete blood count (WBC 7,610/µl, Neutrophils 4,550/µl (59.8%, slightly above the upper limit of normal), Lymphocytes 2,110/µl (27.7%), Hb 12.7 g/dl) and unremarkable inflammatory markers (CRP 0.24 mg/dL, LDH 263 U/L and AST 35 U/L). Urine test was mostly normal with only mild ketonuria noted.

A clinical diagnosis of furuncular lesions, possibly of parasitic origin, was considered, given the clinical features and the recent travel history and cutaneous presentation. The patient was discharged in stable condition with oral amoxicillin-clavulanic acid (50 mg per kg b.i.d.) and corticosteroids (Betamethasone 2 mg b.i.d. for 3 days), antipyretics if needed, and a follow-up plan for outpatient evaluation in the infectious diseases department. The day before follow-up, the mother reported the emergence of a parasite from one of the skin lesions, which was presented for evaluation. Clinical diagnosis of furuncular myiasis was given.

**Patient 3.** A 1-year-old boy, living in Rome, Italy with no significant medical history presented to our hospital in April 2025 for the onset of a cutaneous lesion, with a suspected insect bite acquired during a recent travel to Senegal. The lesion, located in the left scapular region, had appeared approximately six days earlier. Both parents reported similar lesions during the same period. The child was febrile on day four post-onset, with fever resolving spontaneously. Initial treatment consisted of topical gentamicin cream and povidone-iodine applied over 72 hours, and oral amoxicillin-clavulanate initiated on the sixth day due to persistent local inflammation and systemic symptoms.

At presentation, the child was in good general condition. Physical examination revealed a nodular, erythematous, and tender lesion approximately 1 cm in diameter, characterized by a central punctum with purulent discharge and an apparent respiratory pore, raising suspicion for furuncular myiasis. Cardiopulmonary and abdominal examinations were unremarkable. No blood testing was performed.

Surgical evaluation confirmed a tense, mobile, nodular lesion with central punctum. Clinical diagnosis of furuncular myiasis was given.

An occlusive treatment with petroleum jelly was applied over the site to induce larval removal. After 45 minutes, partial extrusion of the larva’s cephalic portion was observed.

**Fig 3:**
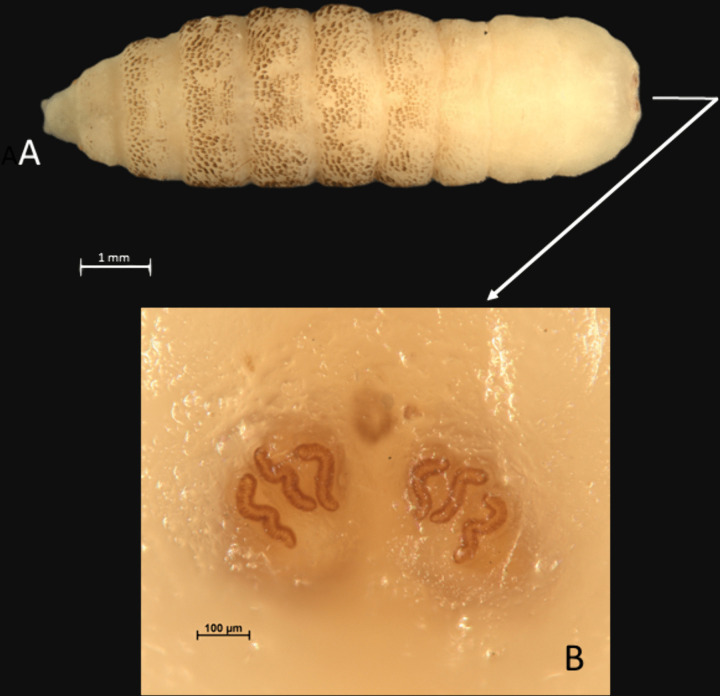
*Cordylobia anthropophaga* larva showing a spine pattern characteristic of the second larval stage (A) and posterior spiracles characteristic of the third larval stage (B). Written informed consent was obtained from the patient’s parents/legal guardians.

Using non-invasive technique, a live and intact larva, approximately 1 cm long and 4 mm wide, was successfully extracted (Fig 3). The specimen was transported alive to the microbiology laboratory in a sterile container for taxonomic identification.

Concomitantly, the mother of the child had undergone surgical removal of a similar larva at Gemelli Hospital (Rome), which was later identified as *C. anthropophaga*.

Post-extraction management included a 7-day course of oral amoxicillin-clavulanate. At follow-up in the Infectious Diseases outpatient clinic 9 days later, the lesion had healed well with no signs of re-infestation or secondary bacterial infection.

### Fly larvae identification

Morphological identification of the larvae was performed at the Laboratory of Parasitology and Entomology of the Istituto Zooprofilattico Sperimentale del Lazio e della Toscana “M. Aleandri”, based on body shape and size, cuticle spine pattern and number and shape of posterior spiracles (Fig 3).

*Patients 1 and 3*: maggots almost cylindrical, 7.5-8.0 mm long. Large *“flame shaped”* spines, with dark brown backwards pointing tips on segments 3–8; thin lines of smaller spines at the posterior margin of the segments 9–11. Two posterior spiracles with three sinusoidal spiracular slits each. The described morphological traits and the geographical origin of the cases allowed identifying the maggots as belonging to the genus *Cordylobia*. Due to spiracular slits shape the species *Cordylobia rhodaini*, characterized by very wavy slits, was ruled out and recovered specimens definitively identified as *C. anthropophaga* [10]. Spines pattern was typical of second stage larvae (Fig 3A), while presence of three spiracular slits and size of the maggots were consistent with third stage larvae (Fig 3B). This mix of characters of the two stages is compatible with second stages at the end of their development and ready to moult to third ones [7].

*Patient 2*: identification based on the morphological study of one of the two recovered larvae, being the other one damaged during its extraction. Maggot cylindrical, 12 mm long. Large “flame shaped” spines, with dark brown tips, on all the segments. Posterior spiracles with three sinusoidal spiracular slits. Morphological features and geographical origin of the case allowed identifying the maggot as third stage larva of *C. anthropophaga*.

## Discussion

Although *C. anthropophaga* myiasis remains primarily limited within sub-Saharan Africa, the number of cases reported in Europe has increased over the past two decades, mainly in travelers returning from endemic regions [9,11-14]. In Italy several cases in adult travelers were reported, including those returning from Senegal and Kenya, with presentations ranging from single to multiple furuncular lesions [11–13,15]. Notably, eyelid myiasis [16] and widespread cutaneous involvement [12] have been described in Italian patients. Other European countries have similarly documented imported cases, with clinical presentations consistent with the classic furuncular pattern [9,14].

Of particular epidemiological significance is a 2018 case report from Livorno, Italy, which described furuncular myiasis in a non-traveling pediatric patient, suggesting the possibility of autochthonous transmission in southern Europe under favorable environmental conditions [17]. This case represents a critical precedent, as it demonstrates that *C. anthropophaga* infestation may occur in non-endemic European regions without direct travel to Africa.

This case series of three pediatric patients diagnosed between April 2024 and April 2025 adds several elements to the European epidemiological picture. First, it represents the largest pediatric case series reported from Italy to date, following the isolated 2018 case [17]. Second, the clustering of cases within 12 months at a single institution may reflect increased travel to endemic areas, greater clinical awareness, or evolving environmental conditions that favor larval survival during travel.

Indeed, beyond the ongoing risk of imported cases due to international travel, rising global temperatures and shifting precipitation patterns could facilitate the geographic expansion of the *tumbu* fly beyond sub-Saharan Africa, potentially enabling autochthonous transmission in previously non-endemic regions [17,18]. This risk is further amplified by the role of animal reservoirs, particularly rodents [19] and dogs [20], which may serve as amplification hosts for *C. anthropophaga* and contribute to its establishment in new geographic areas. The convergence of climate change, increased global mobility, and the presence of competent animal reservoirs underscores the need for a comprehensive surveillance strategy.

From a One Health perspective, enhanced awareness among healthcare providers and travelers, together with proactive surveillance and targeted education, is essential to reduce the risk of local transmission in non-endemic countries. Clinicians should maintain a high index of suspicion for furuncular myiasis in patients with compatible skin lesions after travel to endemic regions, and remain alert to possible autochthonous cases. Addressing the impact of climate change on parasitic infestations such as myiasis is also crucial to protect public health and prevent the establishment and spread of *C. anthropophaga* in non-endemic areas.

**Informed Consent Statement:** At the time of hospitalization, the parents or legal guardians of patients consented to the use of personal data for diagnosis and treatment activities and for future scientific research purposes. Basic demographic and clinical information collected in this study did not infringe upon the rights or welfare of the patient. Data were retrospectively analyzed in line with personal data protection policies, and informed consent was waived because all data were de-identified. In addition, the institutional informed consent form signed upon admission to the Emergency Department states that, as an IRCCS hospital, collected information may be used for scientific publications.

## Supporting information

S1 Dataset Minimal anonymized dataset underlying the results reported in this manuscript. (XLSX)
